# Optimal Placement of Pressure Gauges for Water Distribution Networks Using Entropy Theory Based on Pressure Dependent Hydraulic Simulation

**DOI:** 10.3390/e20080576

**Published:** 2018-08-04

**Authors:** Do Guen Yoo, Dong Eil Chang, Yang Ho Song, Jung Ho Lee

**Affiliations:** 1 School of Civil and Environmental Engineering, The University of Suwon, Gyeonggi-do 18323, Korea; 2 School of Civil, Environmental and Architectural Engineering, Korea University, Seoul 02841, Korea; 3 Department of Civil and Environmental Engineering, Hanbat National University, Daejeon 34158, Korea

**Keywords:** pressure driven analysis, entropy method, water distribution networks, pressure sensor, optimal location

## Abstract

This study proposed a pressure driven entropy method (PDEM) that determines a priority order of pressure gauge locations, which enables the impact of abnormal condition (e.g., pipe failures) to be quantitatively identified in water distribution networks (WDNs). The method developed utilizes the entropy method from information theory and pressure driven analysis (PDA), which is the latest hydraulic analysis method. The conventional hydraulic approach has problems in determining the locations of pressure gauges, attributable to unrealistic results under abnormal conditions (e.g., negative pressure). The proposed method was applied to two benchmark pipe networks and one real pipe network. The priority order for optimal locations was produced, and the result was compared to existing approach. The results of the conventional method show that the pressure reduction difference of each node became so excessive, which resulted in a distorted distribution. However, with the method developed, which considers the connectivity of a system and the influence among nodes based on PDA and entropy method results, pressure gauges can be more realistically and reasonably located.

## 1. Introduction

A water distribution network (WDN) is a civil infrastructure system that transports, distributes, and provides purified water. WDN is a complex system that includes reservoirs, tanks, pipes, pumps, sensors, and valves. Its role is to provide water of adequate quality at a certain flow rate and pressure to consumers. Due to the emergence of the Fourth Industrial Revolution (Industry 4.0) as well as the recent trends in Information and Communications Technologies (ICT) and Advanced Metering Infrastructure (AMI), many data analysis-based studies focusing on big data and artificial intelligence are being actively conducted on WDN operations and management [1]. These studies have the essential goal of acquiring significant values (e.g., new abstracted information from customer behavior) from the analysis of large measurement datasets. As low-cost instruments have become increasingly more prevalent in data collection, more flowmeters and water pressure gauges are installed. However, because operation systems are more complex and maintenance costs are high, installing measurement devices at important points is still critical. In the case of flowmeters, there is a project that installs smart meters that measure usage for every consumer to provide information on daily, weekly, and monthly bases [2]. However, since the measurement method of a pressure gauge is different from a flow meter, it is difficult to integrate measurements with one instrument at present. Therefore, inevitably, a significant additional cost must be invested for pressure measurement. For this reason, the pressure cannot be measured in all positions, so it is installed and operated in a particular location that is deemed important by the person in charge.

For water pressure gauge locations, studies based on steady flow hydraulic analysis are divided into two groups: one aims to calibrate and validate the results of piping network analysis [3,4,5,6,7], while the other concentrates on detecting hydraulic abnormalities like pipe damage and drastic increases in water demand [8,9,10,11,12,13,14]. When piping network analysis results were validated and calibrated, water pressure gauges were installed at points where water pressure could be accurately measured, a large consumer was located, or the physical property of the pipes changed. In contrast, when hydraulic abnormalities were detected at any point, water pressure gauge locations were determined by combining points with the most sensitive reactions to abnormalities (i.e., where abnormalities have the greatest effects).

Walski [3] argued that a WDN could be efficiently managed by measuring water pressure at WDN end-points, as well as other points that have large base demands. Yu and Powell [4] proposed installing gauges at points with high data accuracy. Yoo et al. [9] developed an entropy-based model that use basic data from WDNs to determine pressure metering points, which could represent the operating status of a specific block or simplified multi-regional WDN. In this model, demand quantities are randomly combined by reflecting the variability of demand at each node, and the variations of water pressure, which are obtained from a hydraulic analysis, are quantified. Based on the quantified variations, the entropy-based model classifies optimal pressure metering locations into the nodes that most affect the entire piping network (Giving Entropy), as well as nodes most affected by the network (Receiving Entropy).

An optimal combination of pressure metering points can then be determined by a sensitivity analysis. For the hydraulic analysis model, the U.S. Environmental Protection Agency (EPA) EPANET Version 2 [15] was used, which is a representative Demand Driven Analysis (DDA) software. To detect water loss, Christodoulou et al. [10] optimized sensor arrangements by applying an entropy-based approach and a greedy-search heuristic. Sensor locations were optimized based on the total entropy of the piping network, which was obtained from detection probabilities, sensor combination, and circular detection ranges of water loss of each node. Casillas et al. [11] used a genetic algorithm (GA) as an optimization technique for detecting leak points. The objective function was set to minimize the number of undetected leaks, which occurred randomly and were not isolated in any zone. This method was applied to a WDN in Cyprus. Nejjari et al. [12] analyzed a pressure sensitivity matrix, which considered the uncertainty of leak points, to optimize water pressure gauge locations. They also applied a clustering method to reduce the scale and complexity of gauge arrangement. Steffelbauer and Hanusch [13] considered the uncertainty of demand quantities to identify leak points, and unlike other studies, proposed a cost-benefit function based on the goodness-of-fit statistical test, to quantify the detection efficiency obtained by sensor combinations. Leak detection that considered uncertainties determined sensor locations more accurately but required more sensors than other methods. Like Nejjari et al. [12], Cheng et al. [14] used the K-means clustering method and pressure sensitivity matrix to show the variation (gradient) of node pressure due to nodal outflow. They concluded that the most efficient location of a pressure gauge was a node where the Euclidean distance to the centroid of a cluster was minimized.

Recent studies have tended to set an abnormal condition, such as demand variations, to determine an optimal water pressure gauge location, and commonly utilize hydraulic analysis (variations of nodal pressure). In addition, to quantify pressure variations of nodes, an entropy method was preferred, and optimization and clustering methods were applied to reduce the time required to search for an optimal location. The concept of entropy has been widely used in information theory. In the WDN field, entropy theory has been applied to optimal design and reliability indicators (metrics) calculations [16,17,18,19,20,21,22,23,24,25,26]. Atkinson et al. [21], Creaco et al. [22], Tanyimboh et al. [23], and Raad et al. [24] introduced comparison results among surrogate measures of reliability, resilience, and redundancy including entropy concept. Tanyimboh and Templeman [25] investigated the relationship between the entropy and reliability of WDNs. Santonastaso et al. [26] studied the scaling properties of flow entropy with their size and other topological metrics, which can be easily estimated and applied to constrained optimization procedure. Entropy theory quantifies the uncertainty of a system, and an abnormal state is generally assumed for WDNs. However, although existing studies set abnormal states like change of water supply level at the source (reservoir/tank), demand change at nodes, and pipe damage, the hydraulic analysis for simulating real abnormal conditions was based on DDA, which was not suitable for abnormal conditions.

Other recent studies actively perform a pressure driven analysis (PDA) of water supply pipe systems [27,28,29,30,31,32,33]. PDA considers the supply amount as an unknown quantity because the supply (normally the sum of usage and leaks) changes due to residual heads or water pressure. Existing DDA models such as EPANET2 conduct hydraulic analysis under the assumption that every supply is known and can be attained. So long as the supply was sufficient, the analysis was accurate under normal conditions. However, unrealistic results were produced when an area with a supply shortage was analyzed, the demand changed, or the pipe line was damaged. PDA attempts to solve this problem by introducing the head-outflow relationship (HOR) and using the global gradient algorithm, a numerical method proposed by Todini [34,35].

To determine pressure metering points that could represent WDN operation, this study used the entropy method based on information theory, as well as PDA, which was more suitable for hydraulic analysis purposes in abnormal situations. The main idea is to define failure scenarios based on segment and unintended isolation (UI), and then run a hydraulic simulation and extract the nodal pressures, handle these data points as random variables and apply entropy analysis. In addition, the proposed method was applied to one virtual pipe network and one real pipe network. The priority order for optimal locations was produced, and the result was compared to DDA.

## 2. Materials and Methods

### 2.1. Entropy Theory

The entropy method originated from the part of information theory that analyzes communication signals consisting of numbers, signs, or symbols. Generally, entropy is known as the measure of disorder or uncertainty. Information theory defines entropy as the information capacity of a signal. Entropy can be calculated for any random variable, statistically analyzed, and be used as an objective assessment criterion for information [30]. Entropy theory uses the concepts of marginal entropy, conditional entropy, and transmission to quantify information. The marginal entropy H(X) of a discreet random variable X indicates the amount of information of *X* and is defined by Equation (1) [36]:

(1) $$H\left(X\right)=\overset{n}{\underset{i=1}{\sum }}p\left(x_{i}\right)\ln p\left(x_{i}\right),\;i=1,\;2,\;3,\;\cdots ,\;n$$

where $p\left(x_{i}\right)$ is the occurrence probability of $\left(x_{i}\right)$. If $y_{j}\left(j=1,\;2,\;\cdots ,\;m\right)$ exists and is related to the random variable $x_{i}$, the uncertainty of $x_{i}$ can be reduced by estimating $x_{i}$ from $y_{i}$. Based on this principle, the uncertainly of a random variable X can be estimated based on a given variable Y, as described in Equation (2):

(2) $$H\left(X|Y\right)=-\overset{n}{\underset{i=1}{\sum }}\overset{m}{\underset{j=1}{\sum }}p\left(x_{i},y_{j}\right)\ln p(x_{i}|y_{j})$$

where i and j are the class intervals of the random variables X and Y, respectively, $p\left(x_{i},y_{i}\right)$ is the joint probability of X and Y, and $p(x_{i}|y_{i})$ is the conditional probability of X for a given Y. Consequently, H(X|Y) is the conditional entropy of X for a given Y and indicates the amount of information lost between X and Y [37]. The transmission T(X, Y) between X and Y, which reduces the uncertainty of Y and is the difference between the marginal and conditional entropies, can be calculated through Equations (3) and (4):

(3) $$T\left(X,\;Y\right)=\overset{n}{\underset{i=1}{\sum }}\overset{m}{\underset{j=1}{\sum }}p\left(x_{i},\;y_{j}\right)\ln\left[\frac{p\left(x_{i},\;y_{j}\right)}{p\left(x_{i}\right)p\left(y_{j}\right)}\right]$$

(4) T(X, Y)=H(X)−H(X|Y)

where, i and j are class intervals of the random variables X and Y, respectively, and $p\left(x_{i}\right)$ and $p\left(y_{i}\right)$ are marginal probabilities [38,39]. The concept of marginal entropy is applicable to continuous random variables. If the continuous random variables X and Y are assumed to follow the probability density functions of f(x) and f(y), when the range of X is divided by the interval N of width Δx the marginal entropy of the continuous random variable X is defined by Equation (5), and the conditional entropy and transmission are calculated by Equations (6) and (7), respectively.

(5) $$H\left(X;\Delta x\right)≅\overset{n}{\underset{i=1}{\sum }}f\left(x_{i}\right)\ln f\left(x_{i}\right)\Delta x-ln\Delta x≅-\int_{0}^{\infty }f\left(x\right)\ln f\left(x\right)dx-\ln\Delta x$$

(6) $$H\left(X|Y;\Delta x\right)≅-\int_{0}^{\infty }\int_{0}^{\infty }f\left(x,\;y\right)\ln f\left(x|y\right)dxdy-\ln\Delta x$$

(7) T(X, Y)=H(X)−H(X|Y)

When two continuous random variables X and Y follow a log normal distribution and can reflect a relativeness of the data scales of each variable, the Equations (6) and (7) can be derived by Equations (8) and (9). An interval Δx/x is proportional to ranges of variables, are applied instead of the fixed interval Δx. In this study, when a WDN has an abnormal condition due to pipe failure, the pressure variation at nodes may be zero. In such a case, it is difficult to apply the entropy concept. To solve this problem, Ref. [40] proposed an equation that could apply the entropy concept even if a measured variable includes zero. This study set Δx to 0.01 in Equations (8) and (9), assumed to be a general error tolerance in a hydraulic system. In addition, x was set as the mean value of X. In the case that the variables X and Y contain zero data, marginal entropy, conditional entropy can be calculated as shown in Equations (10)–(12).

(8) $$H\left(X;\Delta x/x\right)≅-\int_{0}^{\infty }f\left(x\right)\ln\left[xf\left(x\right)\right]dx-\ln\left(\Delta x/x\right)$$

(9) $$H\left(X|Y;\Delta x/x\right)≅-\int_{0}^{\infty }\int_{0}^{\infty }f\left(x,\;y\right)\ln\left[xf(x|y)\right]dxdy-\ln\left(\Delta x/x\right)$$

(10) $$H\left(X;\Delta x/x\right)≅-\left(1-k_{x}\right)\ln\left(1-k_{x}\right)-k_{x}\ln\left(k_{x}\right)+k_{x}0.5\ln\left(2\pi e\sigma_{z}^{2}\right)-k_{x}\ln\left(\Delta x/x\right)$$

(11) $$\begin{array}{cc} H\left(X|Y;\Delta x/x\right)≅ & -\left(1-k_{x}-k_{y}+k_{xy}\right)\ln\left(1-k_{x}-k_{y}+k_{xy}\right)-\left(k_{x}-k_{xy}\right)\ln\left(k_{x}-k_{xy}\right)\\ & +\left(1-k_{y}\right)\ln\left(1-k_{y}\right)-\left(k_{y}-k_{xy}\right)\ln\left(k_{y}-k_{xy}\right)+k_{y}\ln\left(k_{y}\right)-k_{xy}\ln\left(k_{xy}\right)\\ & +k_{xy}0.5\ln\left[\left(2\pi e\sigma_{z}^{2}\right)\left(1-\rho_{zw}^{2}\right)\right]-k_{x}\ln\left(\Delta x/x\right) \end{array}$$

(12) T(X, Y;Δx/x)=H(X;Δx/x)−H(X|Y;Δx/x)

In Equation (10)–(12), $\sigma_{z}$ is the standard deviation of z=lnx, $\rho_{zw}$ is the correlation coefficient between z=lnx and w=lny, $k_{x}$ is the probability that X is not zero, $k_{y}$ is the probability that Y is not zero, and $k_{xy}$ is the probability that both X and Y are not zero.

As the entropy method is based on the latest observational data, it is advantageous to use in assessing and maintaining a complete WDN rather than in designing a new WDN. This study quantified the entropy concept to propose node positions as optimal pressure monitoring locations, at which a water pressure change is detected as reasonably as possible and thus the WDN is efficiently maintained.

### 2.2. Pressure Driven Analysis

The analysis of steady flow in a WDN is very complicated because the water supply system consists of various facilities like pipe lines, reservoirs, valves, and booster pumps. The analysis of a WDN is conducted by setting up a basic equation based on fundamental laws of water flow in a pipeline. The basic equation consists of a continuity equation based on the law of conservation of mass, an energy equation based on the law of conservation of energy, and a momentum equation based on Newton’s Second Law of Motion.

Todini and Pilati [41] proposed a gradient method that enabled a very quick hydraulic analysis even for a large-scale pipe network, without matching the continuity of water flow when assuming an initial flow. This method is also referred to as the “Hybrid node-loop method”. Unlike conventional methods such as the Hardy Cross method, the hybrid node-loop method uses node-based determinants for numerical analysis. This method is included in EPANET2 and is widely used in design and management practices. The wide application of the gradient method is attributable to its ability to perform a hydraulic analysis within a few seconds, regardless of the number of analyzable nodes and pipes.

However, the gradient method conducts a hydraulic analysis by setting the usage (demand) of each consumer as a known value. It is assumed that total supply is always equal to usage. Accordingly, the hydraulic analysis can be applied to a basic plan of water supply maintenance or the middle and long-term plan and design of a WDN. On the other hand, when supply is below demand due to water supply restrictions during abnormal conditions (e.g., drought, pipe failure), the pressure head is so low that some areas show chronic poor water supply. Or the water leakage inside a pipe network is so great as to create a big imbalance between usage and supply. Table 1 presents simulated states of WDN analysis according to water usage components and their relationships. For analysis purposes, the global gradient method was developed by modifying the matrix entry of the aforementioned gradient method. Initially introduced by Wu et al. and Todini [35,42], the global gradient method forms an information matrix about head-outflow relation (HOR, [43]) or pressure-leakage relation on the matrix $A_{22}$ (Table 2). Furthermore, nodal demand, which was considered a known value for conventional pipe network analysis, was set to an unknown value as for available water supply or leakage. Results were subsequently calculated using a numerical analysis.

Based on the latest trends in hydraulic analysis, the steady state analysis of a pipe network system can be classified into DDA and PDA. Each item in Table 3 compares DDA and PDA, which are explained as follows:

- DDA: Assuming that demand quantity is known at each point and can always be supplied, continuity and recurrence equations are used to calculate pressure heads at each point. Usage and leakage are input as demand.
- PDA: A numerical analysis that considers the available water supply for each demand point and the leakage of a pipe as pressure-dependent factors. In other words, the variables are determined by a pipe network analysis. Both the head-available water supply and the head-leakage are calculated as unknown values.

This study used the K-NRisk model [44,45]. This model implements a pressure driven-based hydraulic analysis and includes a multi-scenario function that displays multiple scenarios in a single frame.

### 2.3. Pressure Driven Entropy Method (PDEM)

**Figure 1:**
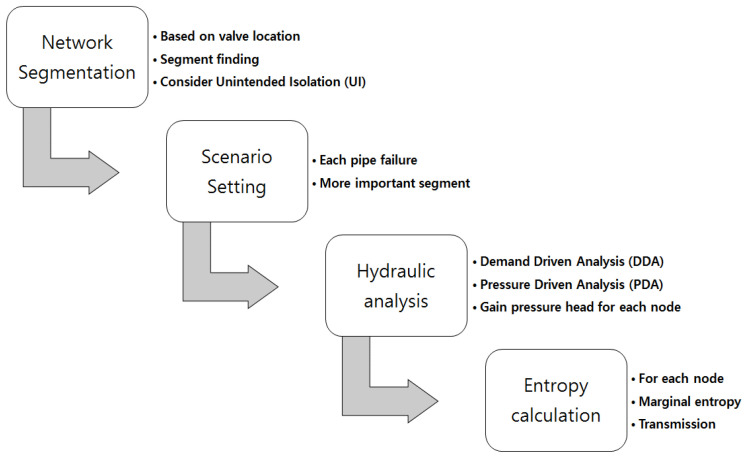
Flow chart of the pressure driven entropy method (PDEM) model.

This study proposes a pressure driven entropy method (PDEM). The procedure to determine optimal pressure measurement is outlined in Figure 1. The specific steps are as follows:

#### 2.3.1. Assumption of a Single Pipe Failure and Scenario Setting

The normal state of a WDN is hydraulically defined as a condition in which water can be supplied at a pressure and a flow rate that causes no discomfort at any time and place. This study assumed that a reservoir was large enough to satisfy every demand within the corresponding WDN. Another assumption was that a pressure change at a node due to a single pipe failure was the main cause of making the abnormal condition in WDNs. Since there is an extremely small likelihood that two or more pipes would simultaneously fail, except during disasters like earthquakes, a single pipe failure was assumed.

**Figure 2:**
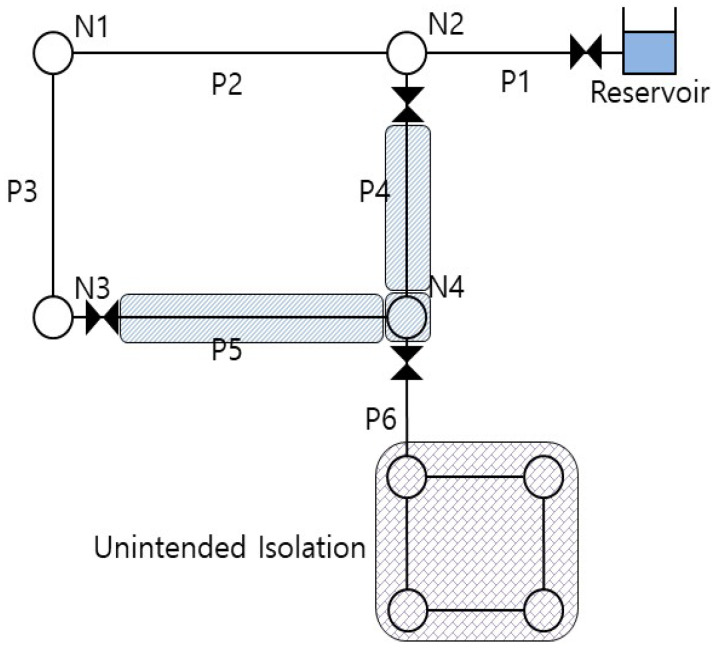
Segment and unintended isolation (unintended isolation (UI)).

When a pipe fails, adjacent water valves should be closed. In a real pipe network, valves are not installed at every possible point but are placed at main points, cost permitting. Accordingly, a zone isolated by valves often includes two or more pipes with demand nodes between them. Walski [46] defined such a zone as the segment of a pipe network; Figure 2 shows an example of such a segment. If P4 or P5 leaks or is fractured, water valves at N1, N3, and N4 must be closed and appropriate measures (e.g., repair or replacement) should be taken. Here, pipes P4 and P5 and node N3 are defined as a single segment because they belong to the same closed zone. There is of course no guarantee that every valve is always properly functioning. Accordingly, when segmentation is considered, it might be necessary to form two or more segments because of a valve malfunction. However, this study did not consider possible valve malfunctions, instead assuming that every valve works perfectly when necessary.

Jun [47] defined unintended isolation (UI) as an isolated area to which the sole passage to the source is unintentionally cut off due to pipe or segment blockage. Although no direct failure occurs in this area, water supply is indirectly shut off due to the failure of another zone. An UI can consist of a single node or a combination of a single node and one or more pipes. As shown in Figure 2, the segment shuts off the lower area connected by P6 from the source, thereby generating an UI.

To represent a typical pipe failure in the hydraulic analysis, this study calculated and applied a practical isolation area formed by UI and the segment. For situations in which there were a few segments within a pipe network, all single pipe failure scenarios were considered. On the other hand, when there were dozens or hundreds of segments, appropriate scenarios were selected by a descending damage scale order (outage, population, interrupted flow rate, etc.) caused by pipe failure. For the virtual pipe network (Ozger’s Network) of this study, both ends of pipes were assumed to be equipped with water valves. A total of 21 segments were considered for the hydraulic analysis. For the real pipe network (Cherry-Hills), out of 80 total segments, 10 segments were assumed to have pipe failure scenarios by considering the real locations of water valves as well as the damage scale.

#### 2.3.2. Hydraulic Analysis for Pipe Failure and Derivation of Water Pressures at Each Node (DDA and PDA)

Basic data like pipe diameter, pipe length, node elevation, basic demand, and the calibrated/corrected frictional coefficients of the pipe were collected to perform a steady-state analysis for each node. To conduct a hydraulic analysis according to the selected pipe failure scenarios, the state of the pipe and the variation at each demand node were set. In other words, the pipe state of a segment was changed from “Open” to “Closed”, and the demand at nodes within the segment was set to “0”.

PDA was performed for each scenario and the pressure and available water supply at each node were obtained. The pressure values of each scenario were arranged in a matrix as shown in Equation (13) and used to calculate entropy. DDA was also conducted and the results were compared with the PDA-based entropy calculations.

(13) $$\left(\begin{array}{cc} \begin{array}{ccc} p_{1,\;1} & p_{2,\;1} & \cdots \\ p_{1,\;2} & p_{2,\;2} & \cdots \\ \vdots & \vdots & \ddots \end{array} & \begin{array}{ccc} p_{j,\;1} & \cdots & p_{n,\;1}\\ p_{j,\;2} & \cdots & p_{n,\;2}\\ \vdots & \ddots & \vdots \end{array}\\ \begin{array}{ccc} p_{1,\;i} & p_{2,\;i} & \cdots \\ \vdots & \vdots & \ddots \\ p_{1,\;m} & p_{2,\;m} & \cdots \end{array} & \begin{array}{ccc} p_{j,\;i} & \cdots & p_{n,\;i}\\ \vdots & \ddots & \vdots \\ p_{j,\;m} & \cdots & p_{n,\;m} \end{array} \end{array}\right)$$

here

j = j-th simulation scenario (j=1, 2, 3, ⋯, n)
n = Number of simulation scenarios
i = i-th node (i=1, 2, 3, ⋯, m)
m = Number of nodes in a pipe network
$p_{j,\;i}$ = Pressure at the i-th node for the j-th simulation scenario.

#### 2.3.3. Calculation of Nodal Entropy and Determination of Pressure Measurement Priority Order

Nodal pressure data obtained at the second stage of each scenario were used to calculate marginal and conditional entropies between nodes, and the transmission was obtained based on the difference between the two entropies. Total entropy was calculated by summing the marginal entropy at a node and the total transmission between the node and another node. When total entropies were arranged in descending order, the priority order for water pressure measurement was determined. Example procedure of entropy calculation is explained in Appendix A.

If a node has a large total entropy, it not only has a lot of information, but also shares a lot of information with other nodes. Any location that can minimize information loss between nodes is considered the best location for monitoring the entire WDN through pressure measurements. If an abnormal accident occurs at a particular point, the optimal location can reasonably detect the accident with minimal loss.

## 3. Results and Discussion

### 3.1. Benchmark Pipe Networks (Ozger’s and Anytown Networks)

**Figure 3:**
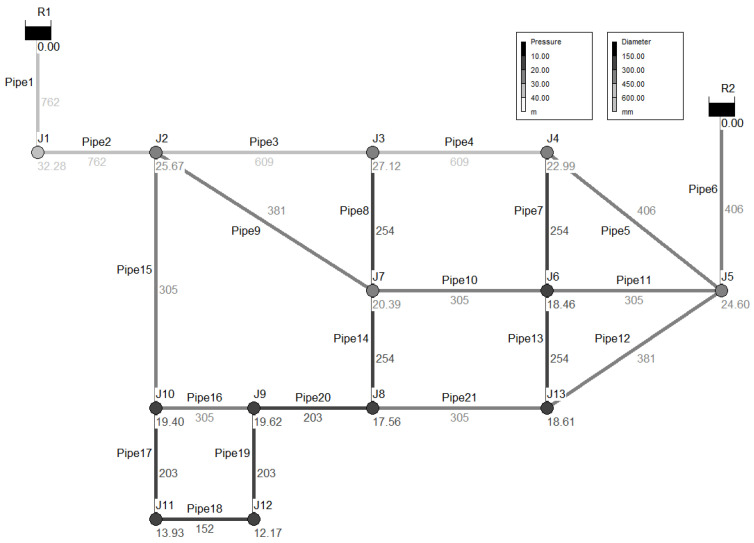
Virtual pipe network of Ozger [48].

Before applying the model to a real pipe network, its applicability was evaluated for two pipe networks with a simple structure. The virtual pipe network, initially proposed by Ozger [48], consisted of 2 reservoirs, 13 nodes, and 21 pipes. Figure 3 illustrates the virtual pipe network while Table 4 and Table 5 present specifications of the nodes and pipes. The other pipe network is tested on a literature benchmark case study of the Anytwon network (Walski et al. [49] and Kapelan et al. [50]). It consisted of 1 source, 2 tanks, 16 nodes, and 34 pipes. Basic input data of the nodes, pipes and nodal demand and source elevation loading condition are adopted “LC1”, proposed by Kapalan et al. [50].

In a normal state, Ozger’s network had a total supply of 3146.4 Cubic Meters per Hour (CMH). Figure 3 shows the pressure distribution of each node under the normal state. For the HOR equation parameters, the minimum pressure head was set to 0 m and the desirable pressure head was set to 15 m, which approximated the minimum reference pressure head (15.3 m) of the Water Supply Facility Criteria [51]. If the minimum reference pressure head was used as the criterion, nodes J11 and J12 did not satisfy the water supply serviceability for consumers, even in a normal state. Pipe failure scenarios assumed that water valves were installed at both ends of the pipes. The failure of 21 total segments was then considered in the hydraulic analysis.

**Figure 4:**
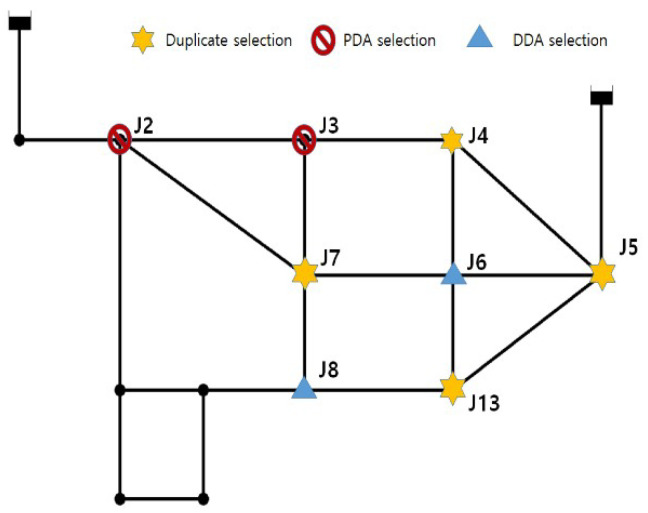
Optimal locations of pressure gauges in each hydraulic analysis (PDA and DDA, Ozger’s Network).

DDA was also conducted and the results were compared with those of the proposed method. Table 6 presents total entropy values of each node in descending order, for production by both PDA and DDA. J4 and J3 had the largest total entropy for PDA, while DDA assigned the largest value to J7 and J13. Although the absolute values of total entropy at each node cannot be compared between PDA and DDA, the difference can be analyzed based on the spatial arrangements of nodes. Figure 4 displays the top six nodes of each hydraulic analysis method; the stars indicate nodes selected by both methods. Of course, the spatial distribution may depend on the number of installation points. However, the DDA-based entropy method selected adjacent points for installing pressure gauges, thereby exhibiting a skewed distribution. In contrast, the PDA-based entropy method selected not only J2, which is adjacent to the upper left source (R1), but also J5, which is near the upper right source (R2). In addition, it exhibited an overall equal distribution of installation points. Generally, when a pipe adjacent to a source in a WDN system has a hydraulic change, the entire system is affected. In this regard, J2 and J5 could be important nodes in terms of connectivity within the relatively simple Ozger’s network, even if no hydraulic result is considered. However, the DDA-based entropy method showed that J2 had the lowest total entropy among all nodes. Accordingly, this method turned out to have low reliability.

The above result is attributable to the impacts of pipe failure in a hydraulic analysis. If a pipe failure did not have a large effect on an entire system, DDA and PDA results were only slightly different. On the other hand, when an entire system was greatly affected by a pipe failure, DDA showed a larger decrease in nodal pressure head than PDA. Table 7 presents the average pressure heads of DDA and PDA and the difference, COV (coefficient of variation) value between them for each pipe failure. The COV (coefficient of variation) is a measure of the dispersion, and is defined as the ratio of the standard deviation to the mean. The COV can also be used as a normalized index to evaluate the system variation. DDA exhibited a larger pressure head decrease in every failure case than PDA did, and the difference was greater in pipes (bold) that affected the entire system. This is because DDA does not change the available water supply based on pressure. Even if the water supply is impossible or limited, DDA assumes that every demand is satisfied, thereby deriving a negative or lower pressure head than in reality. Because of this characteristic, DDA clearly identifies nodes that have a remarkably large effect on an entire system but cannot detect other types of nodes where water supply is sensitive to pressure. In contrast, PDA considers both the one or two nodes that greatly influence an entire system as well as the realistic distribution of pressure heads within the system. Accordingly, the pressure gauge locations are better distributed by PDA.

**Figure 5:**
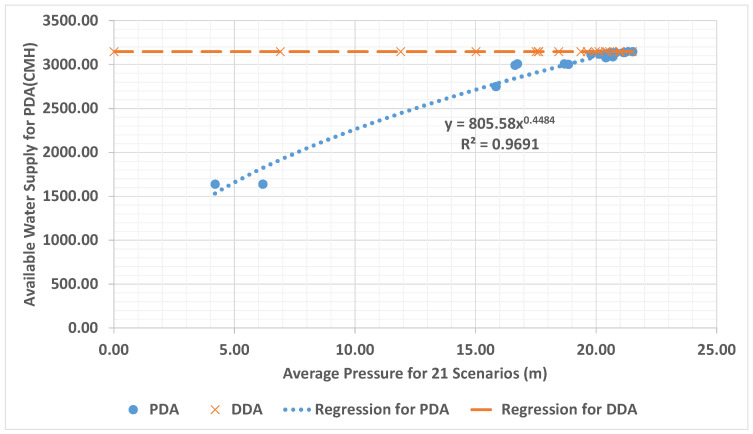
Graph of the average nodal pressure and available water supply based on 21 pipe failure scenarios.

Because the proposed PDEM uses realistic results to calculate information exchange and sharing among nodes, it is more effective than the existing DDA-based method. In addition, PDA results can calculate an available flow rate based on nodal pressure under additional pipe failures. Figure 5 illustrates the average nodal pressures and available water supplies based on pipe failure scenarios. The system pressure head can be lowered to 5 m in a probable pipe failure scenario, limiting the available water supply of the system to 1500 CMH. Accordingly, the water pressure measurement locations might change depending on the impacts and selection range of pipe failure scenarios. In addition, an appropriate abnormal scenario can be set and the corresponding water pressure monitoring locations can be reasonably determined.

The other pipe network is tested on a literature benchmark case study of the Anytwon network to compare with pre-proposed method (Behzadian et al. [52]). In a normal state, this network had a total supply of 6400 Gallon per Minutes (GPM). The nodal pressures range from 47 psi (33 m) to 121 psi (85.1 m), the criterion for the minimum pressure head (15.3 m) was satisfied. The desirable pressure head (m) was set to 45 psi (31.6 m) for the HOR equation parameters. Pipe failure scenarios assumed that water valves were installed at both ends of pipes. The failure of 34 total segments (number of pipes) was then considered in the hydraulic analysis.

**Figure 6:**
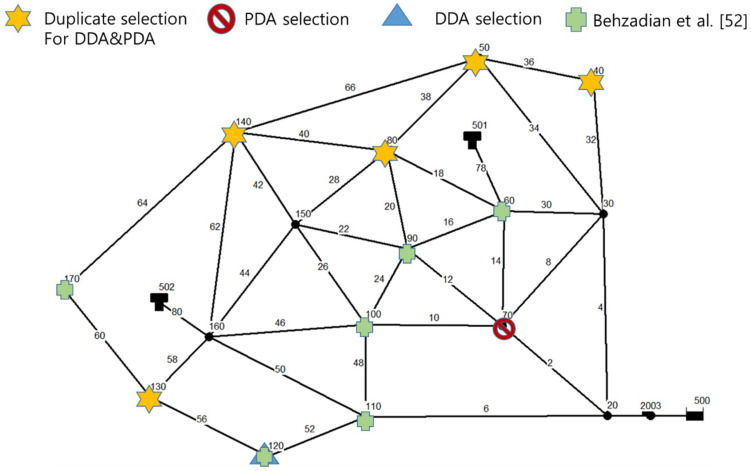
Optimal locations of pressure gauges in each hydraulic analysis (PDA, DDA, and Behzadian et al. [52] in Anytown Network).

Figure 6 illustrates the top six nodes of each hydraulic analysis method; the stars indicate nodes selected by both methods. In addition, the results of previous study proposed by Behzadian et al. [52] is also provided. Behzadian et al. [52] used a multi-objective genetic algorithm with adaptive neural networks (MOGA–ANN) to determine optimal sampling locations for installing pressure loggers when parameter uncertainty is considered. Even though the optimal locations can differ depending on the assumption (parameter uncertainty, used representative scenarios), the relative tendency of location distribution can be compared. In the case of the Anytown network, most priority points (five) of PDA and DDA were selected in duplicate because the network has multiple sources (1 reservoir and 2 tanks) and many loops. This leads to small differences of nodal pressure between normal and abnormal conditions and non-negative pressure even if under DDA simulations. However, for PDA results, the selection of nodal point 70 results in a pressure gauge being evenly distributed throughout the system. A comparison results of Behzadian et al. [52] and PDA show that both results are evenly distributed throughout the system. In the case of Behzadian et al. [52], the optimum pressure gauge placement position appears in the middle of the system, but PDA results are placed at the distal end of the system, relatively.

### 3.2. Real Pipe Network (Cherry Hills Network)

**Figure 7:**
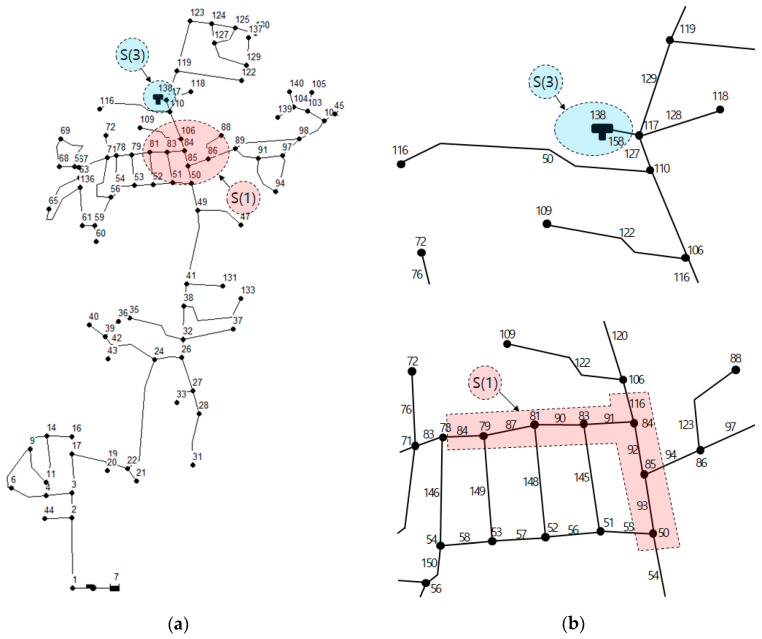
(**a**) Map and (**b**) primary segments of the Cherry Hills Network [9].

The target network is the Cherry Hills WDN in Connecticut, U.S. As shown in Figure 7a, this network consists of 88 nodes, 104 pipes, and 1 tank. The pipe diameters are 6, 8 and 12 inches, respectively, and the pipes are connected in a branched network. The actual locations of water valves were considered to conduct a segment-based analysis. The water valve locations and corresponding segment results were based on the study by Yoo et al. [53]. This network is composed of 80 total subsystems (Segment + UI), and each segment mostly consists of 1–3 pipes with a maximum 7 nodes. Accordingly, the network is divided into small groups; however, the Cherry Hills WDN has many UIs, one of which includes approximately 30 nodes and 40 pipes. If UI occurs, all the demands of the nodes included in the UI are blocked. Accordingly, the effects on the corresponding subsystem would be large. Figure 7b shows the locations and ranges of major segments S(1) and S(3). Among 80 total segments, 10 segments were very affected by water cut-off (Subsystem Important Index, [53]) and were selected as pipe failure scenarios. The Subsystem Importance Index (SII) can quantify the effect of isolating a subsystem on the entire network. The SII is introduced with a range of 0 to 1. A large SII value indicates that the water supply and pressure of an entire water distribution system are decreased severely by the isolation of the corresponding subsystem including the segment and the UI. In case of Cherry Hills Network, we selected the largest 10 subsystems as representative failure scenarios, which have the SII value is over than 0.05.

(14) $$SII_{k}=\frac{Q^{n}-Q_{k}^{es}}{Q^{n}}$$

where,

$SII_{k}$ = SII of Subsystem k
$Q^{n}$ = Total water Demand under normal conditions
$Q_{k}^{es}$ = Total Effective Supply when Subsystem k Isolated

**Figure 8:**
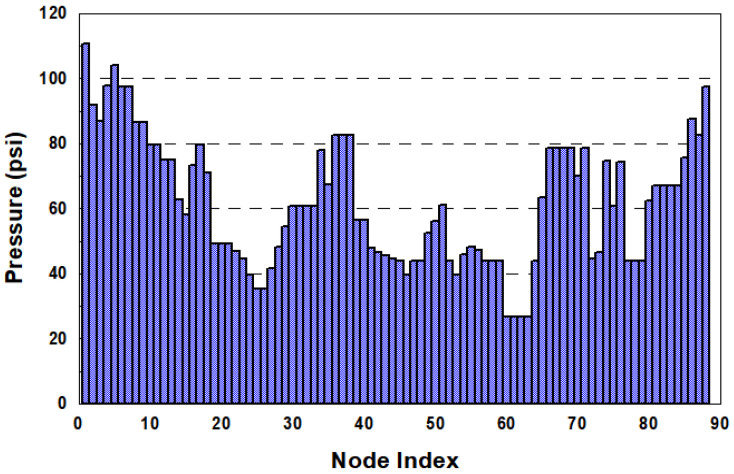
Nodal pressure (psi) distribution of the Cherry Hills Network under normal conditions.

Figure 8 display the pressures of each node under the normal condition of the Cherry Hills Network. The nodal pressures range from 26 psi (18.3 m) to 110 psi (77.3 m), exhibiting a diverse distribution, and the criterion for the minimum pressure head (15.3 m) was satisfied. The desirable pressure head (m) was set to 25 psi (17.6 m), which approximated the lowest pressure within the network.

**Figure 9:**
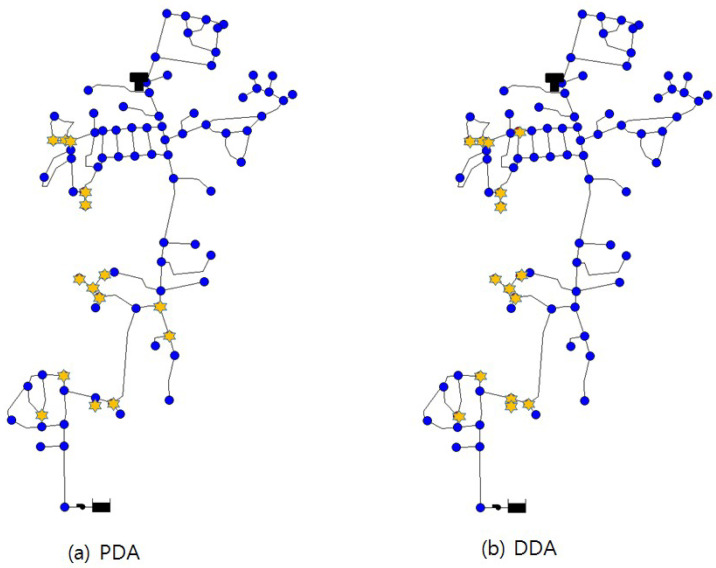
(**a**) PDA and (**b**) DDA-based optimal pressure gauge locations (Cherry Hills Network).

Figure 9 illustrates the results of optimal pressure gauge locations produced by PDA and DDA-based entropy methods, respectively. Among 80 total nodes, 15 nodes were given priority and are marked by stars. As in the Ozger’s network results (Section 3.1), DDA selected adjacent nodes as the installation points and shows a left-skewed distribution within the entire system. Although there were some duplicate nodes between PDA and DDA, PDA showed that pressure gauges should be installed at nodes of the main transmission line, which connects the resources to a tank or end consumers. Accordingly, to derive a final priority order, the PDA-based entropy method considered not only major nodes, but also reasonably reflected the mutual effects among nodes, based on the connectivity of the real network and the hydraulic results.

Table 8 shows total entropy values of top 15 priority nodes in descending order, for production by both PDA and DDA. J36 and J39 had the largest total entropy for PDA, while DDA assigned the largest value to J36 and J40.

Table 9 presents the average pressure, corresponding to the available water supply, amount of water shut-off for each blocked segment, and total amount of water shut-off when a pipe failure occurs, and water supply is cut off for every related segment during repair work; however, water is still supplied to other areas.

## 4. Conclusions

This study proposed a pressure driven entropy method (PDEM) that determines a priority order of pressure gauge locations, which enables the impact of pipe failures to be quantitatively identified. The PDEM utilizes PDA, which is the latest WDN hydraulic analysis method, and the entropy method, which can quantify information exchange and sharing between two datasets. The conventional DDA was problematic in determining the locations of pressure gauges, attributable to results of the hydraulic analysis. If one or two pressure gauges were installed, the DDA-based entropy method had no difficulty selecting nodes that had a large effect on a system. This was because in an abnormal state, DDA caused a much larger nodal pressure decrease than PDA did. However, the pressure reduction difference in each node became so excessive that the priority order of installation was determined not by the hydraulic relation among nodes, but by the absolute pressure of each node, which resulted in a distorted distribution. Accordingly, if the proposed method, which considers the connectivity of a system and the influence among nodes based on PDA results, is adopted, pressure gauges can be more realistically and reasonably located. This study applied the proposed method to two simple benchmark networks and a real pipe network. As the proposed method also considers UI and segments that are real damage ranges of a single pipe failure, it is expected to be utilized to design and maintain real WDNs.

Even though PDA has some advantages compared to DDA (i.e., more realistic than the fixed demand modeling), PDA also has some uncertainties such as the limitation in defining the head-outflow relationship (HOR) [53] so that this point should be considered.

In case of Cherry Hills network, the location of pressure gauges are not evenly distributed in the network because we determined priority locations according to total entropy value among entire nodes. In further study, we can divide the whole system into some sub-areas (e.g., district meter area in water supply system) before apply our proposed method. In addition, we can incorporate with the clustering method (e.g., k-means) or spatial distribution function as a constraint. If we have some prior information, such as existing sensor (tank level, flowmeter) and valve locations, it can be used as constraints in the proposed method. Future work also needs to simultaneously apply more scenarios incorporating uncertainty by using an optimization method. Beyond a single pipe failure, additional abnormal situations (multiple pipe failures due to disasters such as earthquakes, drastic demand increases due to drought or firefighting use, and other factors including uncertainty) also need to be simulated to analyze the impact and significance on a system.

## Figures and Tables

**Table 1:** Simulated states for pipe network analysis according to water use relation.

Simulated State	Water Use Relation	Example
Normal condition	Supply = Total usage (demand)	Middle and long-term construction plan and design of water distribution networks (WDNs)
	Supply = Usage + Leakage	Current pipe network analysis considering usual leakage
Abnormal condition	Supply = Usage + Leakage	Pipe network analysis considering the dominant effect of leakage
	Supply < Usage	Shortage of supply due to drought or demand change
	Supply < Usage + Leakage	Pipe failure due to various causes

**Table 2:** Gradient Method and Global Gradient Method.

	Gradient Method	Global Gradient Method
Matrix Equation	$\left[\begin{array}{cc} A_{11} & A_{12}\\ A_{21} & 0 \end{array}\right]\left[\begin{array}{c} Q\\ H \end{array}\right]=\left[\begin{array}{c} -A_{10}\;H_{0}\\ -q_{0} \end{array}\right]$	$\left[\begin{array}{cc} A_{11} & A_{12}\\ A_{21} & A_{22} \end{array}\right]\left[\begin{array}{c} Q\\ H \end{array}\right]=\left[\begin{array}{c} -A_{10}\;H_{0}\\ -q^{*} \end{array}\right]$
Notation (different)	$q_{0}=\left[n_{n},\;1\right]$ known nodal demands	$q^{*}=\left[1,\;n_{n}\right]$ vector of actual demands and leakages for pressure deficient nodes
		$A_{22}=\left[n_{n},\;n_{n}\right]$ diagonal matrix for pressure-dependent (head-outflow, pressure-leakage relation) nodes with elements
Notation (common)	$Q=\left[n_{p},\;1\right]$ unknown pipe discharge	
	$H=\left[n_{n},\;1\right]$ the unknown nodal heads	
	$H_{0}=\left[n_{t}-n_{n},\;1\right]$ known nodal heads	
	$A_{11}=\left[n_{p},\;n_{p}\right]$ diagonal matrix for pipes and pumps	
	$A_{12}$, $A_{21}=\left[n_{p},\;n_{t}\right]$ topological incidence matrix that defines the pipe and node connectivity	
	$A_{10}=\left[n_{t}-n_{n},\;1\right]$ topological incidences for known-head nodes	
	$n_{t}$ = total number of nodes, $n_{n}$ = number of unknown-head nodes	
	$n_{p}$ = total number of links	

**Table 3:** Comparison of Demand Driven Analysis (DDA) and pressure driven analysis (PDA).

Analysis Method	DDA	PDA
Application	Normal operation	Normal/Abnormal operation
Reliability of normal state analysis	High	High
Reliability of abnormal state analysis	Low	High
Assumption	Demand is always satisfied	Supply at each node and leakage of a pipe network are affected by the pressure heads of the node and pipe network.
Disadvantage	Unrealistic results like negative pressures may be derived from a hydraulic analysis of abnormal state.	A relation (head-outflow relation (HOR)) is needed between the pressure head and supply at each node, as well as between the water pressure and leakage of a pipe network.

**Table 4:** Specifications of nodes of the virtual pipe network.

ID	Elevation (m)	Demand (CMH)	Type	Degree of Node (DoN)
1	27.43	0	Junction	2
2	33.53	212.4	Junction	4
3	28.96	212.4	Junction	3
4	32	640.8	Junction	3
7	29.56	640.8	Junction	4
6	31.39	684.0	Junction	4
5	30.48	212.4	Junction	4
13	33.53	0	Junction	3
8	31.39	327.6	Junction	3
9	32.61	0	Junction	3
10	34.14	0	Junction	3
11	35.05	108	Junction	2
12	36.58	108	Junction	2
20 (R1)	60.96	-	Reservoir	1
21 (R2)	60.96	-	Reservoir	2

**Table 5:** Specifications of pipes of the virtual pipe network.

ID	Start Node	End Node	Length (m)	Diameter (mm)	Roughness, C (H-W)
P1	20 (J1)	1	609.6	762	130
P2	1	2	243.8	762	128
P3	2	3	1524	609	126
P4	3	4	1127.76	609	124
P5	4	5	1188.72	406	122
P8	3	7	944.88	254	116
P10	7	6	883.92	305	112
P7	4	6	762	254	118
P9	2	7	1676.4	381	114
P11	6	5	883.92	305	110
P13	6	13	762	254	106
P12	13	5	1371.6	381	108
P15	2	10	944.88	305	102
P14	7	8	822.96	254	104
P16	10	9	579	305	100
P20	9	8	883.92	203	92
P21	8	13	944.88	305	90
P17	10	11	487.68	203	98
P18	11	12	457.2	152	96
P19	9	12	502.92	203	94
P6	21 (J2)	5	640	406	120

**Table 6:** Priority order of optimal installation and total entropy in each hydraulic analysis (PDA and DDA in Ozger’s Network).

Rank	PDA		DDA	
	Node	Total Entropy	Node	Total Entropy
1	J4	13.72	J7	17.29
2	J3	12.95	J13	17.12
3	J5	12.9	J4	17.08
4	J2	12.87	J5	17.03
5	J7	12.67	J6	16.85
6	J13	12.33	J8	16.48
7	J6	12.28	J3	16.3
8	J8	11.64	J10	16.29
9	J1	11.51	J9	15.92
10	J9	10.96	J1	14.24
11	J10	10.73	J12	13.86
12	J12	9.57	J11	13.57
13	J11	9.34	J2	12.12

**Table 7:** Hydraulic analysis results (PDA and DDA) according to pipe failure scenarios.

Failure Pipe	Average Pressure Head (m)			Available Water Supply (PDA, CMH)
	DDA (1)	PDA (2)	Difference (2-1)	
P1	−58.34 (UR *)	**4.19**	**62.53**	**1637.3**
P2	−51.45 (UR *)	**6.17**	**57.62**	**1637.3**
P3	11.89	15.84	3.95	2749.65
P4	17.6	18.68	1.08	3007.01
P5	21.01	21.17	0.16	3136.55
P6	15.01	16.63	1.62	2991.76
P7	20.45	20.64	0.19	3134.69
P8	20.38	20.58	0.2	3134.21
P9	17.51	18.85	1.34	3002.03
P10	20.99	21.15	0.16	3136.9
P11	20.11	20.39	0.28	3121.67
P12	19.37	19.78	0.41	3115.84
P13	21.01	21.16	0.15	3136.72
P14	20.55	20.8	0.25	3132.71
P15	6.9	16.73	9.83	3007.58
P16	19.64	20.14	0.5	3119.11
P17	17.62	20.4	2.78	3077.88
P18	20.97	21.14	0.17	3136.37
P19	18.45	20.69	2.24	3089.7
P20	21.5	21.51	0.01	3146.14
P21	19.96	20.6	0.64	3099.34
COV ** (%)	194.8	8.6	-	-

* UR-Unrealistic Negative Pressure; ** COV (coefficient of variation) = standard deviation (σ)/mean (μ) × 100.

**Table 8:** First 15 priorities of optimal installation and total entropy in each hydraulic analysis (PDA and DDA in Cherry Hills Network).

Rank	DDA			PDA		
	Node	Total Entropy	Average Pressure Head (psi)	Node	Total Entropy	Average Pressure Head (psi)
1	Junc 36	144.7	70.9	Junc 36	145.6	72.9
2	Junc 40	144.7	70.9	Junc 39	145.6	72.9
3	Junc 39	143.1	70.9	Junc 40	145.6	72.9
4	Junc 60	141.3	56.1	Junc 42	139.7	72.9
5	Junc 59	140.9	56.1	Junc 16	137.8	92.0
6	Junc 5	140.6	77.8	Junc 60	137.5	58.2
7	Junc 42	140.1	70.9	Junc 11	137.4	102.9
8	Junc 20	139.3	83.2	Junc 26	137.3	64.0
9	Junc 68	139.3	77.8	Junc 27	137.3	79.2
10	Junc 67	139.2	77.8	Junc 5	137.0	79.9
11	Junc 19	139.1	83.2	Junc 68	136.9	79.9
12	Junc 16	139.0	90.1	Junc 59	136.5	58.2
13	Junc 22	138.8	78.6	Junc 20	136.3	85.1
14	Junc 78	138.7	43.2	Junc 22	136.1	80.5
15	Junc 11	138.7	101.0	Junc 67	136.0	79.9

**Table 9:** Hydraulic analysis results for each scenario for the Cherry Hills Network.

Segment	Average Pressure after Segment Isolation		Available Water Supply	Amount of Water Cut-off in Each Segment (GPM *)	Total Amount of Water Cut-off (GPM)
	DDA	PDA	(PDA, GPM *)		
S1	58.62	58.62	970.38	2.25	2.25
S2	73.87	73.87	811.11	161.52	161.52
S3	74.09	74.09	899.64	72.99	72.99
S4	83.26	83.26	551.64	420.99	420.99
S5	61.79	61.79	836.31	136.32	136.32
S6	61.72	61.72	848.82	123.81	123.81
S7	45.64	46.36	804.97	162.33	167.66
S8	61.64	61.64	893.85	78.78	78.78
S9	61.77	61.77	908.46	64.17	64.17
S10	14.75	34.59	861.91	0	110.72

* Gallons per minute.

## Appendix A

Calculate the entropy for a virtual network (Ozger’s Network).

**Table A1:** Step 1. Hydraulic result under a normal condition of PDA.

Node ID	Demand	Head	Pressure
	CMH	m	m
J1	0	59.71	32.28
J2	212.4	59.2	25.67
J3	212.4	56.08	27.12
J4	640.8	54.99	22.99
J5	212.4	55.08	24.6
J6	684	49.85	18.46
J7	640.8	49.95	20.39
J8	327.6	48.95	17.56
J9	0	52.23	19.62
J10	0	53.54	19.4
J11	108	48.98	13.93
J12	108	48.75	12.17
J13	0	52.14	18.61

**Table A2:** Step 2. Head under an abnormal condition for each scenarios of PDA.

ID	S.1	S.2	S.3	S.4	S.5	S.6	S.7	S.8	S.9	S.10	S.11	S.12	S.13	S.14	S.15	S.16	S.17	S.18	S.19	S.20	S.21	Ave.	ST.D
J1	7.82	33.53	33.07	32.80	32.25	31.42	32.28	32.30	32.54	32.30	32.24	32.24	32.28	32.30	32.45	32.33	32.35	32.29	32.33	32.30	32.29	31.22	5.38
J2	1.72	1.72	26.78	26.40	25.62	24.45	25.66	25.69	26.03	25.69	25.60	25.60	25.66	25.69	25.90	25.74	25.76	25.68	25.74	25.70	25.68	23.45	7.24
J3	6.41	6.41	12.39	30.33	26.84	22.43	27.31	27.52	26.13	27.10	27.16	27.22	27.13	27.15	26.98	26.96	27.21	27.13	27.21	26.99	27.30	24.35	6.88
J4	3.71	3.71	9.39	12.19	22.58	16.37	23.37	23.06	21.58	22.93	23.19	23.28	23.04	23.01	22.71	22.75	23.08	23.01	23.10	22.81	23.31	19.63	6.54
J5	12.42	12.42	16.34	18.33	25.12	14.05	24.78	24.57	23.10	24.52	25.43	25.48	24.76	24.58	24.24	24.31	24.68	24.62	24.71	24.38	25.11	22.28	4.51
J6	4.34	4.34	10.00	12.80	18.58	11.61	15.94	16.77	13.41	17.92	14.48	15.75	17.38	18.76	17.47	17.74	18.59	18.48	18.65	17.94	18.30	15.20	4.40
J7	5.09	5.09	12.63	17.40	20.48	14.92	18.86	18.24	12.69	20.92	18.13	18.13	19.87	21.03	19.32	19.61	20.53	20.42	20.60	19.82	19.93	17.32	4.74
J8	3.74	3.74	10.76	13.96	17.77	11.53	16.46	16.07	11.68	17.82	15.96	13.69	17.78	15.04	13.73	15.07	17.82	17.62	18.02	15.86	12.41	14.12	4.13
J9	2.59	2.59	18.75	19.32	19.95	17.59	19.58	19.49	18.53	20.00	19.40	18.79	19.97	19.20	6.82	13.15	20.71	19.88	21.94	22.68	18.51	17.12	5.83
J10	1.07	1.07	19.15	19.44	19.67	17.70	19.43	19.36	18.79	19.73	19.28	18.86	19.70	19.17	5.16	23.19	21.50	19.71	20.80	21.68	18.68	17.29	6.37
J11	0.13	0.13	13.88	14.20	14.51	12.81	14.27	14.21	13.64	14.55	14.15	13.75	14.53	14.03	3.46	14.45	5.10	14.99	12.06	16.54	13.57	11.85	4.96
J12	0.00	0.00	12.07	12.46	12.85	11.07	12.59	12.52	11.86	12.89	12.46	12.02	12.87	12.32	2.00	8.80	9.18	12.36	5.02	14.93	11.82	10.10	4.43
J13	5.51	5.51	10.72	13.25	18.95	10.19	17.80	17.78	15.06	18.51	17.65	12.39	20.14	18.08	17.23	17.66	18.76	18.64	18.83	17.94	20.91	15.79	4.49

**Table A3:** Step 3. Calculate a difference of head between a normal and an abnormal condition for each nodes of PDA.

ID	S.1	S.2	S.3	S.4	S.5	S.6	S.7	S.8	S.9	S.10	S.11	S.12	S.13	S.14	S.15	S.16	S.17	S.18	S.19	S.20	S.21	Ave.	ST.D
																						Wo/Zero Data	
J1	24.47	1.25	0.79	0.52	0.03	0.86	0.00	0.02	0.26	0.02	0.05	0.04	0.00	0.02	0.17	0.05	0.06	0.01	0.05	0.02	0.01	1.37	5.30
J2	23.96	23.96	1.11	0.73	0.05	1.22	0.01	0.02	0.36	0.02	0.07	0.07	0.01	0.02	0.23	0.07	0.09	0.01	0.07	0.02	0.00	2.48	7.15
J3	20.71	20.71	14.73	3.21	0.28	4.69	0.19	0.40	0.99	0.02	0.04	0.10	0.01	0.03	0.14	0.16	0.08	0.01	0.09	0.13	0.18	3.19	6.69
J4	19.29	19.29	13.60	10.80	0.41	6.62	0.38	0.07	1.41	0.06	0.20	0.29	0.05	0.02	0.28	0.24	0.09	0.02	0.11	0.18	0.32	3.51	6.46
J5	12.18	12.18	8.26	6.27	0.52	10.55	0.18	0.03	1.50	0.08	0.83	0.88	0.16	0.02	0.36	0.29	0.08	0.02	0.10	0.22	0.51	2.63	4.33
J6	14.12	14.12	8.46	5.66	0.12	6.85	2.52	1.69	5.06	0.54	3.98	2.71	1.08	0.30	0.99	0.72	0.13	0.02	0.19	0.52	0.16	3.33	4.34
J7	15.30	15.30	7.76	2.99	0.09	5.48	1.53	2.15	7.71	0.53	2.26	2.26	0.52	0.64	1.07	0.78	0.14	0.03	0.21	0.57	0.47	3.23	4.63
J8	13.83	13.83	6.80	3.60	0.21	6.03	1.10	1.49	5.88	0.26	1.60	3.87	0.22	2.52	3.83	2.49	0.26	0.05	0.46	1.70	5.15	3.58	4.01
J9	17.03	17.03	0.87	0.30	0.33	2.03	0.04	0.13	1.09	0.38	0.22	0.83	0.35	0.42	12.80	6.47	1.09	0.25	2.32	3.06	1.11	3.25	5.43
J10	18.33	18.33	0.25	0.04	0.27	1.70	0.03	0.04	0.61	0.33	0.12	0.54	0.30	0.23	14.24	3.79	2.10	0.31	1.40	2.28	0.72	3.14	5.91
J11	13.80	13.80	0.05	0.27	0.58	1.12	0.34	0.28	0.30	0.62	0.22	0.18	0.60	0.10	10.47	0.52	8.83	1.06	1.87	2.61	0.36	2.76	4.60
J12	12.17	12.17	0.10	0.29	0.68	1.10	0.42	0.35	0.31	0.72	0.29	0.15	0.70	0.15	10.17	3.37	2.99	0.19	7.15	2.76	0.35	2.69	4.06
J13	13.10	13.10	7.89	5.36	0.34	8.42	0.81	0.83	3.55	0.10	0.97	6.22	1.53	0.53	1.38	0.95	0.15	0.03	0.22	0.67	2.30	3.26	4.16

**Table A4:** Step 4. Exchange a constant data to a log data.

ID	S.1	S.2	S.3	S.4	S.5	S.6	S.7	S.8	S.9	S.10	S.11	S.12	S.13	S.14	S.15	S.16	S.17	S.18	S.19	S.20	S.21	Ave.	ST.D
J1	3.20	0.22	−0.24	−0.65	−3.41	−0.15	−6.91	−4.07	−1.36	−3.96	−3.10	−3.12	−6.21	−4.02	−1.78	−2.92	−2.73	−4.51	−2.98	−3.86	−5.12	−2.75	2.34
J2	3.18	3.18	0.10	−0.31	−2.98	0.20	−5.12	−3.91	−1.03	−3.82	−2.69	−2.72	−4.96	−3.86	−1.45	−2.63	−2.44	−4.51	−2.70	−3.69	−5.30	−2.26	2.42
J3	3.03	3.03	2.69	1.17	−1.27	1.55	−1.64	−0.92	−0.01	−3.86	−3.35	−2.33	−4.61	−3.51	−1.97	−1.85	−2.47	−4.61	−2.41	−2.04	−1.70	−1.29	2.37
J4	2.96	2.96	2.61	2.38	−0.88	1.89	−0.96	−2.69	0.34	−2.83	−1.63	−1.24	−2.92	−4.07	−1.26	−1.43	−2.39	−3.91	−2.23	−1.71	−1.15	−0.87	2.21
J5	2.50	2.50	2.11	1.84	−0.65	2.36	−1.73	−3.41	0.41	−2.50	−0.19	−0.13	−1.81	−3.86	−1.02	−1.22	−2.48	−4.14	−2.25	−1.52	−0.67	−0.76	2.09
J6	2.65	2.65	2.13	1.73	−2.15	1.92	0.93	0.53	1.62	−0.61	1.38	1.00	0.08	−1.20	−0.01	−0.33	−2.06	−3.82	−1.67	−0.66	−1.85	0.11	1.78
J7	2.73	2.73	2.05	1.10	−2.41	1.70	0.42	0.76	2.04	−0.63	0.81	0.82	−0.66	−0.45	0.07	−0.25	−1.95	−3.61	−1.55	−0.57	−0.77	0.11	1.68
J8	2.63	2.63	1.92	1.28	−1.56	1.80	0.10	0.40	1.77	−1.34	0.47	1.35	−1.53	0.92	1.34	0.91	−1.35	−2.90	−0.77	0.53	1.64	0.49	1.53
J9	2.84	2.84	−0.14	−1.20	−1.12	0.71	−3.30	−2.01	0.08	−0.96	−1.53	−0.19	−1.04	−0.87	2.55	1.87	0.09	−1.37	0.84	1.12	0.11	−0.03	1.63
J10	2.91	2.91	−1.38	−3.19	−1.31	0.53	−3.69	−3.32	−0.50	−1.11	−2.15	−0.61	−1.21	−1.45	2.66	1.33	0.74	−1.19	0.34	0.83	−0.32	−0.44	1.92
J11	2.62	2.62	−2.90	−1.32	−0.55	0.11	−1.07	−1.26	−1.22	−0.47	−1.52	−1.70	−0.51	−2.34	2.35	−0.66	2.18	0.05	0.63	0.96	−1.02	−0.24	1.61
J12	2.50	2.50	−2.34	−1.24	−0.39	0.10	−0.87	−1.04	−1.18	−0.33	−1.25	−1.90	−0.36	−1.91	2.32	1.21	1.10	−1.69	1.97	1.01	−1.05	−0.14	1.56
J13	2.57	2.57	2.07	1.68	−1.07	2.13	−0.21	−0.19	1.27	−2.34	−0.04	1.83	0.42	−0.64	0.32	−0.05	−1.93	−3.47	−1.50	−0.40	0.83	0.18	1.66

**Table A5:** Step 5. Calculate each $k_{x}$, $k_{y}$ and $k_{xy}$ at the nodes.

ID	# of Non-Zero of Scenario	$k_{x}$	$k_{xy}$-J1	$k_{xy}$-J2	$k_{xy}$-J3	$k_{xy}$-J4	$k_{xy}$-J5	$k_{xy}$-J6	$k_{xy}$-J7	$k_{xy}$-J8	$k_{xy}$-J9	$k_{xy}$-J10	$k_{xy}$-J11	$k_{xy}$-J12	$k_{xy}$-J13
J1	21	1.00 *	-	1.00 **	1.00	1.00	1.00	1.00	1.00	1.00	1.00	1.00	1.00	1.00	1.00
J2	21	1.00	1.00	-	1.00	1.00	1.00	1.00	1.00	1.00	1.00	1.00	1.00	1.00	1.00
J3	21	1.00	1.00	1.00	-	1.00	1.00	1.00	1.00	1.00	1.00	1.00	1.00	1.00	1.00
J4	21	1.00	1.00	1.00	1.00	-	1.00	1.00	1.00	1.00	1.00	1.00	1.00	1.00	1.00
J5	21	1.00	1.00	1.00	1.00	1.00	-	1.00	1.00	1.00	1.00	1.00	1.00	1.00	1.00
J6	21	1.00	1.00	1.00	1.00	1.00	1.00	-	1.00	1.00	1.00	1.00	1.00	1.00	1.00
J7	21	1.00	1.00	1.00	1.00	1.00	1.00	1.00	-	1.00	1.00	1.00	1.00	1.00	1.00
J8	21	1.00	1.00	1.00	1.00	1.00	1.00	1.00	1.00	-	1.00	1.00	1.00	1.00	1.00
J9	21	1.00	1.00	1.00	1.00	1.00	1.00	1.00	1.00	1.00	-	1.00	1.00	1.00	1.00
J10	21	1.00	1.00	1.00	1.00	1.00	1.00	1.00	1.00	1.00	1.00	-	1.00	1.00	1.00
J11	21	1.00	1.00	1.00	1.00	1.00	1.00	1.00	1.00	1.00	1.00	1.00	-	1.00	1.00
J12	21	1.00	1.00	1.00	1.00	1.00	1.00	1.00	1.00	1.00	1.00	1.00	1.00	-	1.00
J13	21	1.00	1.00	1.00	1.00	1.00	1.00	1.00	1.00	1.00	1.00	1.00	1.00	1.00	-

* $k_{x}$ of J1 = 21/21 = 1.00; ** $k_{xy}$-J2(the probability that both J1 and J2 are not zero) = (21 + 21)/(21 + 21) = 1.00.

**Table A6:** Step 6. Calculate the correlation coefficient between nodes using the log data at the Step 4.

ID	J1	J2	J3	J4	J5	J6	J7	J8	J9	J10	J11	J12	J13
J1	1.00	0.96	0.81	0.79	0.77	0.60	0.64	0.63	0.67	0.55	0.38	0.40	0.61
J2	0.96	1.00	0.85	0.85	0.82	0.69	0.71	0.65	0.66	0.56	0.43	0.45	0.67
J3	0.81	0.85	1.00	0.95	0.85	0.73	0.77	0.75	0.45	0.30	0.19	0.25	0.78
J4	0.79	0.85	0.95	1.00	0.96	0.78	0.77	0.73	0.43	0.30	0.18	0.23	0.82
J5	0.77	0.82	0.85	0.96	1.00	0.79	0.77	0.73	0.46	0.33	0.14	0.19	0.86
J6	0.60	0.69	0.73	0.78	0.79	1.00	0.97	0.77	0.24	0.10	-0.02	0.09	0.86
J7	0.64	0.71	0.77	0.77	0.77	0.97	1.00	0.88	0.33	0.18	-0.01	0.10	0.89
J8	0.63	0.65	0.75	0.73	0.73	0.77	0.88	1.00	0.52	0.35	0.03	0.16	0.89
J9	0.67	0.66	0.45	0.43	0.46	0.24	0.33	0.52	1.00	0.96	0.68	0.77	0.41
J10	0.55	0.56	0.30	0.30	0.33	0.10	0.18	0.35	0.96	1.00	0.78	0.81	0.25
J11	0.38	0.43	0.19	0.18	0.14	-0.02	-0.01	0.03	0.68	0.78	1.00	0.90	-0.01
J12	0.40	0.45	0.25	0.23	0.19	0.09	0.10	0.16	0.77	0.81	0.90	1.00	0.09
J13	0.61	0.67	0.78	0.82	0.86	0.86	0.89	0.89	0.41	0.25	-0.01	0.09	1.00

**Table A7:** Step 7. Calculate the marginal entropy of each nodes and the transmission between nodes.

ID	J1	J2	J3	J4	J5	J6	J7	J8	J9	J10	J11	J12	J13	Total Entropy
J1	7.19 *	1.23 **	0.53	0.50	0.44	0.22	0.26	0.26	0.29	0.18	0.08	0.09	0.23	11.51
J2	1.23	7.82	0.66	0.65	0.55	0.33	0.35	0.28	0.29	0.19	0.10	0.12	0.30	12.87
J3	0.53	0.66	8.04	1.13	0.66	0.38	0.45	0.42	0.11	0.05	0.02	0.03	0.47	12.95
J4	0.50	0.65	1.13	8.08	1.31	0.46	0.45	0.38	0.10	0.05	0.02	0.03	0.57	13.72
J5	0.44	0.55	0.66	1.31	7.73	0.49	0.45	0.38	0.12	0.06	0.01	0.02	0.69	12.90
J6	0.22	0.33	0.38	0.46	0.49	7.81	1.42	0.46	0.03	0.00	0.00	0.00	0.68	12.28
J7	0.26	0.35	0.45	0.45	0.45	1.42	7.71	0.73	0.06	0.02	0.00	0.00	0.77	12.67
J8	0.26	0.28	0.42	0.38	0.38	0.46	0.73	7.72	0.15	0.07	0.00	0.01	0.78	11.64
J9	0.29	0.29	0.11	0.10	0.12	0.03	0.06	0.15	7.69	1.28	0.31	0.45	0.09	10.96
J10	0.18	0.19	0.05	0.05	0.06	0.00	0.02	0.07	1.28	7.82	0.47	0.53	0.03	10.73
J11	0.08	0.10	0.02	0.02	0.01	0.00	0.00	0.00	0.31	0.47	7.51	0.83	0.00	9.34
J12	0.09	0.12	0.03	0.03	0.02	0.00	0.00	0.01	0.45	0.53	0.83	7.46	0.00	9.57
J13	0.23	0.30	0.47	0.57	0.69	0.68	0.77	0.78	0.09	0.03	0.00	0.00	7.71	12.33

* The marginal entropy of node 1 = $-\left(1-1\right)\times \ln\left(1-1\right)-1\ln\left(1\right)+1\times 0.5\times \ln(2\times \pi \times \exp\left(1)\times 2.34^{2}\right)-)\times 2\times \ln\left(\frac{0.01}{1.37}\right)=7.19$; ** The transmission entropy between node 1 and node 2 = The marginal entropy – The conditional entropy; The marginal entropy of node 1 = 7.19; The conditional entropy = $-\left(1-1-1+1\right)\times \ln\left(1-1-1+1\right)-\left(1-1\right)\times \ln\left(1-1\right)+\left(1-1\right)\times \ln\left(1-1\right)-\left(1-1\right)\times \ln\left(1-1\right)+1\times \ln\left(1\right)-1\times \ln\left(1\right)+1\times 0.5\times \ln\left\{\left(2\times \pi \times \exp\left(1\right)\times 2.34^{2}\right)\times \left(1-0.96^{2}\right)\right\}-1\times \ln\left(\frac{0.01}{1.37}\right)=5.96$; Then transmission entropy = 7.19−5.96=1.23.
